# CDC Operational Guidance for Investigating Locally Acquired Mosquito-Transmitted Malaria — United States, 2026

**DOI:** 10.15585/mmwr.rr7501a1

**Published:** 2026-05-21

**Authors:** Nikhil Ranadive, Joel L.N. Barratt, Ellen M. Dotson, John E. Gimnig, Audrey E. Lenhart, Rebecca S. Levine, Kimberly E. Mace, Peter D. McElroy, Brian H. Raphael, Dean Sayre, Laura C. Steinhardt, Alice C. Sutcliffe, Seymour G. Williams, Alison D. Ridpath

**Affiliations:** ^1^Epidemic Intelligence Service, CDC; ^2^Division of Parasitic Diseases and Malaria, National Center for Emerging and Zoonotic Infectious Diseases, CDC; ^3^Department of Environmental and Occupational Health Sciences, University of Washington, Seattle, Washington; ^4^Department of Pathology and Laboratory Medicine, Emory University School of Medicine, Atlanta, Georgia

## Abstract

**During 2023, 10 cases of locally acquired mosquito-transmitted (autochthonous) malaria were reported to CDC from four U.S. states after a 20-year period with no autochthonous cases. These cases highlight that although the United States eliminated malaria transmission in the early 1950s, the country remains susceptible to malaria reintroduction:**

**This report provides operational guidance to local, state, tribal, territorial, and Federal public health responders on public health investigation and response to a suspected autochthonous malaria case in the United States. The guidance in this report updates the 2006 guidance (CDC. Locally acquired mosquito-transmitted malaria: a guide for investigations in the United States. Atlanta, GA: US Department of Health and Human Services, CDC; 2006) and is based on CDC subject-matter expertise, outbreak response experience from 2023, and targeted literature review:**

**Investigations of suspected autochthonous malaria cases often raise public concern and can require substantial public health resources. Public health investigations of these cases should include epidemiologic, entomologic, and laboratory components and frequently require coordination among local, state, and Federal jurisdictions. This report provides an outline of the essential actions for such investigations conducted by describing the components of the investigation and providing resources and tools to assist public health personnel at the local, state, and Federal level:**

## Introduction

Malaria is a mosquito-transmitted disease that causes substantial global morbidity and mortality. Despite having eliminated malaria from the United States in the early 1950s (*1*), the country remains susceptible to reintroduction because local mosquitos capable of transmission could bite someone who acquired malaria parasites outside of the United States and spread it to persons who have not traveled. The risk varies by geography, vector ecology, climate, travel pattern, and public health capacity. Approximately 2,000 cases of malaria and an average of seven malaria-related deaths are reported annually to CDC, nearly all of which are diagnosed among travelers returning from countries where malaria is endemic (*2*,*3*). Since 1972, the annual number of cases reported among U.S. civilians has increased (*2*,*3*), and conditions in much of the country remain suitable for local transmission, particularly in areas where *Anopheles* mosquitoes are indigenous (*4*–*7*). After elimination (interruption of local transmission), numerous small outbreaks of geographically limited locally acquired mosquito-transmitted (autochthonous) malaria have occurred, with 30 reported outbreaks during 1980–2003 (*8*). This period was followed by 2 decades without any reported autochthonous malaria cases (*9*). However, in 2023, local malaria transmission was again observed, with 10 cases of autochthonous malaria reported across four states (*9*–*12*), during a year that had the highest number of imported malaria cases (n = 2627) since elimination (*13*).

Local transmission of malaria in the United States is a significant public health concern because the majority of U.S. residents lack protective immunity against malaria, rendering persons susceptible to severe illness and death if infected. Mosquitoes capable of transmitting malaria are present across most of the United States. Human malaria is primarily caused by four protozoan species*: Plasmodium falciparum*, *Plasmodium vivax*, *Plasmodium ovale*, and *Plasmodium malariae* and is transmitted through bites from infective female mosquitoes of the genus *Anopheles*. *Plasmodium* infection in humans can result in a spectrum of disease, ranging from asymptomatic parasitemia (typically among persons with naturally acquired immunity who live in endemic settings) to severe illness and death. Although any *Plasmodium* species can cause severe malaria, it is most associated with *P. falciparum*. Patients with malaria infections might first experience nonspecific signs and symptoms (e.g., fever, chills, headache, generalized weakness, myalgia, vomiting, and diarrhea) (*14*). Progression to severe malaria can manifest with altered mental status, circulatory failure, shock, multisystem organ failure, and death (*14*). Although *P. falciparum* is the most common species of malaria globally and has caused autochthonous cases in the United States, most U.S. outbreaks were due to infection by *P. vivax* (*8*). One factor that possibly favors *P. vivax* transmission in the United States is that *P. vivax* gametocytes (the form of the parasite that is infectious to mosquitoes) develop in the peripheral blood earlier in the disease process (often even before symptom onset) than *P. falciparum* gametocytes (*15*,*16*). In addition, *P. falciparum* infection has a shorter incubation period and typically progresses to illness more quickly, prompting earlier health care–seeking, diagnosis, and treatment. In contrast, *P. vivax* symptoms are generally less severe, and infectious persons can go undetected for longer periods before diagnosis and treatment, thereby extending the person’s period of potential transmission to local *Anopheles* spp.

The CDC’s National Malaria Surveillance System (NMSS) (*2*) was established in the 1940s to monitor and evaluate progress toward malaria elimination in the United States. NMSS is designed to detect cases of malaria diagnosed in the United States and classify each case as having been acquired from domestic or international exposures. In addition, surveillance is used to identify and characterize unusual cases of public health importance. These cases include those acquired within the United States from blood (e.g., transfusion- or transplant-transmitted infections) or local mosquito exposures, malaria-infected patients with poor clinical outcomes, and antimalarial treatment failures. Malaria surveillance data guide public health response activities that attempt to detect and interrupt local transmission and prevent reintroduction. This report updates the 2006 CDC guidance for public health officials responding to possible cases of locally acquired mosquito-transmitted malaria in the United States based on experience from the 2023 outbreak investigations and new methodologies and tools developed during the responses.

## Methods

Updated guidance and tools for investigating autochthonous malaria in the United States were based on the authors’ (staff members in the Malaria Branch, Entomology Branch, and Laboratory Sciences and Diagnostics Branch, all within CDC’s Division of Parasitic Diseases and Malaria) expert knowledge of the subject matter, published literature, and experience assisting health departments responding to locally transmitted malaria during the 2023 outbreaks. An informal literature review was conducted in PubMed and Google Scholar for documents including dates during 2006–2025; the search terms included “local transmission”, “autochthonous”, “malaria”, “elimination”, and “pre-elimination”. Guidance documents and grey literature from European CDC, Australia, and World Health Organization (WHO) were also assessed. All articles and guidance documents were reviewed by authors for additional methods or interventions that could be applicable to the U.S. setting. Stakeholder engagements were conducted with public health department staff members who responded to locally transmitted cases during 2023 (in Arkansas, Florida, Maryland, and Texas) to ensure completeness and accuracy of these public health activities. Consultations were also conducted with staff members from health departments who had not had recent experience investigating a potential locally acquired malaria case to ensure feasibility. These public health strategies were presented during the 2024 Council for State and Territorial Epidemiologists and American Society of Tropical Medicine and Hygiene annual conferences for additional input and feedback. Differences across jurisdictions related to resources, infrastructure, and mosquito ecology were factored into developing the guidance. This guidance will be updated as new evidence, methodology, or interventions become available.

### Guidance for Routine Malaria Surveillance and Case Investigations

All patients with clinically suspected malaria should be tested for *Plasmodium* infection as soon as possible to ensure correct diagnosis, guide treatment (*17*), and prevent onward transmission. Because malaria is a notifiable disease, laboratory tests in which *Plasmodium* is detected are reported to state, territorial, local, or tribal health departments according to jurisdictional reporting requirements, often electronically. CDC and the Council for State and Territorial Epidemiologists define a confirmed malaria case as the detection of any *Plasmodium* spp. in any person by microscopy or polymerase chain reaction (PCR) (*18*). Cases are investigated by health departments using standardized case report forms (*19*) submitted to NMSS (*2*). Malaria case investigations explore risk factors for acquiring malaria, such as a history of travel to endemic settings, previous malaria infections, and a history of blood transfusions or organ transplants. Detailed processes for routine malaria surveillance and case investigations, including eliciting this information through patient interviews, medical record reviews, and completion of malaria case report forms have been described previously (*20*).

CDC classifies malaria cases as imported (travel-associated), induced (from bloodborne exposure), introduced (from local mosquitoes in an area where malaria is not a regular occurrence), or cryptic (an isolated case that cannot be epidemiologically linked to additional cases and for which an epidemiologic investigation does not identify the mode of acquisition) (*2*,*18*). Nearly all malaria cases in the United States are classified as imported after a routine case investigation (*2*).

## Guidance for Enhanced Investigation for Suspected Autochthonous Malaria Transmission

Malaria cases with no recent travel to an endemic area (*21*,*22*) typically require an enhanced investigation. Enhanced investigation refers to public health surveillance activities beyond routine case investigation. This updated guidance focuses specifically on enhanced investigations for suspected autochthonous cases of malaria. Guidance for investigations in which malaria transmission is suspected from blood transfusion or other bloodborne exposures has been described previously (*23*,*24*).

An enhanced investigation of a suspected autochthonous malaria case typically involves collaboration with epidemiology, entomology, and laboratory staff members at the local, state, and Federal level; and detailed epidemiologic interviews, entomologic assessments, and advanced laboratory testing, as indicated, to assess potential local transmission. An overview of activities that might be included in an enhanced investigation is provided (Box 1). Although these steps are listed sequentially, in practice, many are performed in parallel. Each investigation is unique, might vary by resources available at the jurisdiction, and might be closed without implementing all the activities described. In addition, investigation and response activities and duration might need to be adapted by *Plasmodium* species, temperature, vector ecology, uncertainty in exposure site, and human mobility. CDC subject matter experts are available to assist health departments with epidemiologic investigations, vector surveillance and control activities, laboratory testing including molecular surveillance, and outreach activities. The CDC Malaria Hotline, a clinical service that is always available, can be consulted to discuss any case, especially those which might be the result of local mosquito transmission or from bloodborne or unknown exposure (Box 2).

BOX 1 **Enhanced investigation checklist for autochthonous malaria outbreaks in the United States**

- **Implement epidemiologic investigation.**
  - Verify the diagnosis in a reference laboratory.
  - Conduct in-depth patient interviews.
  - Review surveillance data to identify epidemiologic links.
  - Perform retrospective and prospective case-finding activities.
- **Conduct vector surveillance and control activities.**
  - Initiate vector surveillance activities.
  - Initiate vector control activities.
  - Perform vector laboratory analyses.
- **Perform molecular surveillance of *Plasmodium* species parasites.**
  - Conduct genotyping.
  - Assess for strain relatedness.
- **Conduct community and partner outreach.**
  - Alert the medical community.
  - Educate local community members.
  - Engage local media outlets.
- **Determine whether the outbreak has ended (no new cases identified for at least 8 weeks).**

BOX 2 **Malaria outbreak consultative and laboratory services offered by CDC**

**Clinical and epidemiologic services**

Contact the CDC toll-free malaria hotline (770-488-7788 or 855-856-4713, Monday–Friday, 9 a.m.–5 p.m. Eastern Standard Time; or 770-488-7100 after hours and on weekends and Federal holidays) to notify CDC about an autochthonous malaria outbreak and request a consultation on diagnosis and treatment of malaria from a CDC malaria clinician.

**Entomologic services**

- Vector surveillance and control: request advice and guidance at malarialab@cdc.gov for *Anopheles* species surveillance and control methodologies.
- Vector laboratory analysis: request services at malarialab@cdc.gov for detection of malaria parasites in mosquitoes and for molecular confirmation of mosquito species.

**Laboratory services**

- Malaria diagnostic services: contact dpdx@cdc.gov to request diagnostic services for malaria (e.g., digital or physical review of microscopic slides and polymerase chain reaction testing).
- Malaria molecular surveillance: contact malarialab@cdc.gov to request molecular surveillance services (e.g., genotyping, strain-typing, and resistance marker testing).

### Enhanced Investigation Task A: Implement Epidemiologic Investigation

The purpose of an epidemiologic investigation for a malaria case possibly acquired from local transmission is to ascertain 1) when and where the person was likely infected by local mosquitoes, 2) when and where the person with malaria could have been infectious to local mosquitoes, and 3) whether transmission is ongoing. This process involves verifying the diagnosis, conducting an in-depth patient interview, reviewing surveillance data, and undertaking case-finding activities.

#### Verify the Diagnosis in a Reference Laboratory

Although cases of malaria suspected of being autochthonous are typically already laboratory confirmed at a clinical laboratory, it is important to verify the diagnosis of *Plasmodium* infection at a public health reference laboratory. Diagnosis can be verified at a public health laboratory via microscopy, although for cases of epidemiologic concern, acquiring whole blood specimens for PCR confirmatory testing is advised. PCR is more sensitive than microscopy and can help confirm the *Plasmodium* species when morphological characterization is inadequate (*25*). In addition, PCR can differentiate *Plasmodium* spp. from *Babesia* spp., which cause the disease babesiosis. Endemic to the United States, babesiosis can resemble malaria clinically and microscopically (*26*). The timelines for exposure (mosquito bite), incubation (time from infectious bite to symptom onset), and infectivity (when gametocytes are present in peripheral blood) can be estimated from the *Plasmodium* species detected. Although most public health laboratories provide reference laboratory services for malaria, CDC is available to assist jurisdictions that lack that capacity by providing malaria diagnostic services to assist with both microscopic examination of blood smears and PCR testing (Box 2).

#### Conduct In-Depth Patient Interviews

In addition to the standard malaria investigation, patients without a recent history of travel to a setting where malaria is endemic require a more detailed interview. Their travel history might need to be revisited, because previously undisclosed travel often is revealed through additional patient or family interviews or, when legally authorized and feasible, review of official travel records such as passports. Questions pertaining to additional risk factors for malaria, such as occupation, bloodborne exposures, outdoor activities, and proximity to other sick persons (e.g., household members or household guests) (*20*) might help determine how the patient acquired malaria (Box 3). After other exposure routes of malaria (travel-associated or bloodborne) have been ruled out, detailed questions about time spent outside, participation in outdoor activities (particularly in the evenings, when *Anopheles* mosquitoes bite), housing instability or homelessness, and any other possible exposures to mosquitoes might be asked (Box 4). Patients should be assessed for exposure risks covering the 2–4 weeks before symptom onset, depending on the incubation period of the *Plasmodium* species, and about mosquito exposures and outdoor activity up until the time of malaria treatment initiation to determine the possibility of ongoing transmission.

BOX 3 **Topics to cover in detailed interview for enhanced investigation of patients with malaria**

**Ask about lifetime and recent travel to a malaria-endemic country.**

- Identify specific dates the patients were in a malaria-endemic county and the areas visited.
- If patients previously lived in a malaria-endemic country, when did they immigrate to the United States or to another nonendemic country?

**Ask about previous diagnoses of malaria (in lifetime), or previous unexplained febrile illness after international travel.**

- If yes, list specific dates and if (and what) treatment was received.
- If diagnosed with a relapsing species (*Plasmodium vivax* or *Plasmodium ovale*), did the patients receive antirelapse therapy (e.g., a full treatment course of primaquine or 1 dose of tafenoquine)?

**Ask whether the patients experienced any blood exposures (e.g., via blood transfusions, organ transplants, needlestick injuries, unsafe needle sharing, or home tattoos).**

**Ask whether before the illness began, if there were any visitors, household members, or coworkers who were sick with malaria or another febrile illness.**

**Ask whether the patients have been in an area where *Babesia* parasites are transmitted and whether they had a recent tick bite.**

**Ask whether the patients have recently slept outdoors and whether they currently or have recently experienced housing instability or homelessness.**

**Ask about additional details from the medical records including past medical history (especially immunocompromising conditions, asplenia, and pregnancy status), recent hospitalizations, and medical procedures.**

BOX 4 **Outdoor exposure questionnaire for epidemiologic investigation of patients with malaria**

**Characterize locations where the patients spent time, especially outdoors, while at home, work, or other settings.**

- What are the patients’ regular routines (at home, school, or work)?
- How do they get to and from school or work?
- When does school or work occur (e.g., during the evenings or during the daytime only)?
- What are the routine activities done at home, school, or work?
- Who do they interact with at home, school, or work?

**Characterize the types of outdoor activities the patients participate in, especially in the evenings. For example, do they**

- Frequent any parks or outdoor spaces?
- Participate in dog walking or outdoor sporting events?
- Participate in outdoor cooking such as barbecues?
- Partake in gardening, lawn mowing, or yard work?
- Frequent areas outside of their residence?

**Characterize where patients experiencing housing instability or homelessness spend their time. For example, do they**

- Sleep in the same place every night?
- Stay in any shelters?
- Stay with friends or family?

**Characterize exposure history of patients who have traveled domestically.**

- Did they travel within the United States during the previous 2 months?
- Where did they go?
- What types of activities did they do?
- How did they spend the evenings and early mornings?

**Determine additional risk factors not obtained during initial investigation.**

**Ask whether any household members, coworkers, extended family, friends, or other persons with whom the patients spent time been sick or had fever in the past month.**

**Ask whether the patients recall any mosquito bites?** (If yes, obtain additional details.)

**Ask whether the patients took any medications before their illness?** (If the patients took doxycycline or other antimalarial medications, consider extending the period for questions as above.)

#### Review Malaria Surveillance Data to Identify Epidemiologic Links

Health departments should consider conducting a review of malaria case surveillance data to search for other cases in geographic or temporal proximity to the patient to ascertain if potential epidemiological links exist. This process typically involves evaluating cases in persons infected with an unknown or the same *Plasmodium* species who were diagnosed within approximately 1–2 months before the patient’s symptom onset and who were located ≤5 miles (≤8 km) from the patient’s likely place of exposure. This time frame accounts for the mosquito lifecycle, the human incubation period, and potential delays in diagnosis (Figure) (Table). Health departments should then determine if any of these cases have a relevant link to the patient, or if any patients have unclear travel histories requiring reinvestigation. Health departments should consider confirming the travel histories of patients for any open malaria cases as soon as possible. Obtaining any remaining whole blood specimens collected at the time of malaria diagnosis from patients identified in geographic and temporal proximity to the case of concern is ideal for facilitating further molecular investigation (see Enhanced Investigation Task C: Perform Molecular Surveillance of *Plasmodium* Parasites).

**FIGURE:**
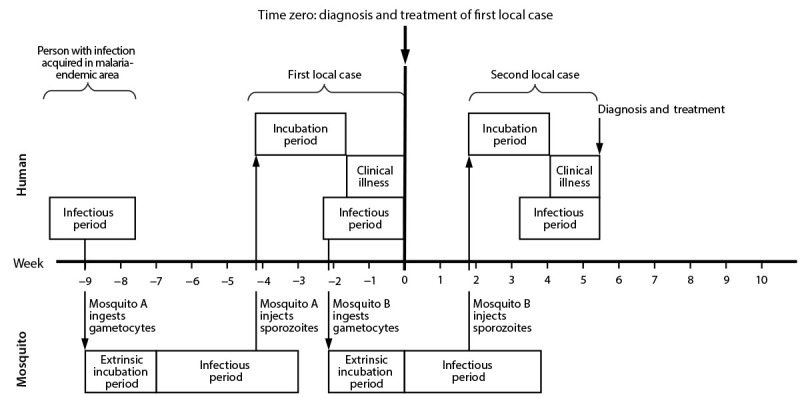
Temporal progression of malaria outbreak for humans* and mosquitoes^†^ * Human incubation and infectious periods vary depending on species and host factors. This figure is based on *Plasmodium vivax* estimations. The average incubation period is 1–2 weeks for *Plasmodium falciparum* and 2–3 weeks for *P. vivax*; however, persons from areas in which malaria is endemic with partial immunity can be asymptomatic. Human infectious period occurs when gametocytes (sexual stage of the parasites) are circulating in the blood. *P. vivax* gametocytes can appear before symptom onset, and *P. falciparum* gametocytes usually appear approximately 7–10 days after symptom onset. *P. vivax* gametocytes are usually killed with treatment, but *P. falciparum* gametocytes might be found in the blood weeks after treatment, depending on the treatment regimen. ^†^ Mosquito extrinsic incubation period is the time from when a mosquito ingests gametocytes from an infected human to when mature sporozoites have developed that can be injected through a mosquito bite into another human, approximately 8–16 days. Mosquito infectious period is the time from after completion of the extrinsic incubation period to the end of its lifespan, approximately 4 weeks.

**TABLE:** Public health response activities and duration after report of a potential locally acquired mosquito-transmitted malaria case

Public health response activity	Description	Duration*
Human case public health vigilance period^†^		
Review malaria surveillance data	Identify malaria cases associated with the same *Plasmodium* species (or unknown species) ≤5 miles (≤8 km) from the likely place of exposure of local case. • Confirm travel histories. • Evaluate potential epidemiologic links. • Obtain available whole blood specimens.	Retrospective review: cases with illness onset 4–8 weeks before time zero
		Prospective review: cases with illness onset at least 8 weeks from time zero
Perform retrospective and prospective case finding	Identify persons with symptoms consistent with malaria for possible testing. • Surrounding hospitals and clinics (could use syndromic surveillance methods) • Household members • Other persons with similar outdoor exposures	Retrospective case-finding: 4–8 weeks before time zero
		Prospective case-finding: at least 8 weeks from time zero
Health care provider education	Provide access to training on malaria diagnosis and treatment.	At least 8 weeks from time zero
Diagnosis and treatment support	Depending on local capacity, assistance from state public health laboratories or CDC might be necessary to ensure prompt malaria diagnostic testing is available.	At least 8 weeks from time zero
Community and partner outreach	Provide awareness to the community on • Risk for malaria • Prevention measures • Malaria symptoms and when to seek care	At least 8 weeks from time zero
Mosquito public health vigilance period^†^		
Vector surveillance and testing of collected mosquitoes	Conduct the following tasks within a radius of ≥1 mile (≥1.6 km) around the suspected site of transmission: • Trap and collect mosquitoes. • Identify mosquito species and test for *Plasmodium*. • Locate vector larval habitats. • Assess for insecticide resistance.	At least 6 weeks from time zero
Vector control	Implemented over a radius of ≥1 mile (≥1.6 km) around the site of suspected transmission • Adulticiding • Larval source management	At least 6 weeks from time zero

* Time zero represents the diagnosis or treatment date of the most recent local case.

^†^ Mosquito and human vigilance periods start when a potential local case is diagnosed or treated. Human case vigilance period continues for at least 8 weeks from the end of the human infectious period (2 weeks for mosquito extrinsic incubation period plus 4 weeks for mosquito infectious period plus 2 weeks for human incubation period). Mosquito vigilance period continues for at least 6 weeks after the last known case is diagnosed (2 weeks for extrinsic incubation period plus 4 weeks for the mosquito’s expected lifespan). Delays in care-seeking or diagnosis might occur; therefore, health departments could decide to continue the vigilance period beyond 8 weeks after a potential local case is identified.

#### Perform Retrospective and Prospective Malaria Case–Finding Activities

In collaboration with health care providers and facilities, health department staff members should consider starting case-finding activities as soon as possible to identify if other persons were infected with malaria locally. Case-finding activities should aim to identify patients who might have already interacted with the health care system but were misdiagnosed because of a lack of relevant travel history, as well as those who become ill after identification of the initial case, to ensure that all receive a correct diagnosis and treatment. The specific activities employed, and the geographic range of their application might be based on epidemiologic investigation findings, feasibility, population density, local ecological factors, and the flight range of local vectors.

Retrospective and prospective case-finding most often starts at surrounding hospitals and clinics (particularly facilities that serve high-risk populations, such as persons experiencing housing instability or homelessness). Syndromic surveillance, using the Electronic Surveillance System for the Early Notification of Community-Based Epidemics (ESSENCE) platform, has proved to be helpful in certain jurisdictions for malaria case–finding as well as for other vectorborne disease outbreak responses (*27*,*28*). Example ESSENCE code for malaria case–finding, to identify patients with potentially compatible signs or symptoms of clinical malaria (fever plus thrombocytopenia or anemia) for whom diagnostic testing can be considered, is available from CDC upon request. Retrospective case-finding should focus on patients examined at health care facilities with unexplained fever during the 1–2 months before the initial or index patient's symptom onset (*8*,*20*). The health care providers of patients identified through this process can be contacted to assess their patients for ongoing symptoms, alternative diagnoses, and to determine if malaria testing is needed.

Prospectively, household members of the index case, as well as others with the same outdoor exposures as the index case, with a current history of fever or chills should be referred for prompt evaluation. Public health authorities should notify health care facilities in the area regarding the need for heightened clinical suspicion for malaria, beginning with facilities in closest proximity to the patient’s residence or likely area of exposure. Patients arriving at local health care facilities with signs and symptoms and laboratory findings consistent with malaria should be evaluated for malaria. Health departments might engage health care facilities and providers to assist with testing, treatment, and reporting additional suspected cases. Depending on local capacity, assistance from state public health laboratories or CDC might be necessary to ensure that prompt malaria diagnostic testing is available.

A period of public health vigilance for additional human cases should continue from the date of diagnosis or treatment for the patient with the last known local case and continue for at least 8 weeks. This 8-week threshold reflects an average period for the parasite lifecycle in the mosquito (extrinsic incubation), adult mosquito lifespan, and the human incubation period (Table) (Figure). Depending on the temperature, vector species and abundance, and delays for care-seeking and diagnosis, this period might be extended beyond 8 weeks, or the vigilance period could be decreased. If a new human case is identified, the case vigilance period would start over.

### Enhanced Investigation Task B: Undertake Vector Surveillance and Control Activities

#### Initiate Vector Surveillance Activities

Detection of locally acquired malaria could require initiating vector surveillance for *Anopheles* mosquitoes, particularly in settings where no routine surveillance exists. The goal of vector surveillance is to identify the species involved in transmission and to assess the impact of implemented control measures. These vector assessment activities are typically conducted by local or state mosquito control programs, but this will vary depending on the capacity of local jurisdictions.

Activities for vector surveillance include trapping and collecting mosquitoes, identifying mosquito species, locating vector larval habitats, assessing for insecticide resistance, and measuring vector control activity effectiveness on mosquito abundance. Vector activities should be conducted around the suspected site of transmission of an autochthonous case beginning within at least a 1-mile (1.6 km) radius as an initial operational perimeter, a typical *Anopheles* flight range, with expansion based on species, ecology, and uncertainty in exposure location.

Evidence for how to best implement vector surveillance and control is limited; local and state mosquito control programs could customize strategies to the specific geography and species of malaria-competent *Anopheles* mosquitoes inhabiting their area (*29*). Methods for mosquito trapping and collection include overnight collections with CDC light traps (with or without carbon dioxide as lure) and aspirating from resting sites such as resting boxes (prefabricated simulated resting sites), trash bins, or sheds; mosquito yields from these trapping methods will vary by species (*30*). Potential larval sites can be assessed by dipping (sampling aquatic habitats) to detect the presence of *Anopheles* larvae. Vector larval habitats, particularly areas of standing water, can also be identified using aerial photo analysis and remote sensing techniques (*31*). Mosquito insecticide resistance levels can be measured using in vivo methods such as the CDC bottle bioassay or WHO tube test (*32*) in addition to molecular, sequencing, and biochemical methods. Vector control experts should identify *Anopheles* species either morphologically or by using molecular methods (as described in Enhanced Investigation Task C: Perform Molecular Surveillance of *Plasmodium* Parasites). Control activity effectiveness can be measured via the effects on various entomological parameters that include vector density, presence of *Plasmodium* DNA in the abdomen of trapped mosquitoes, and the presence of *Plasmodium* sporozoites in mosquito salivary glands (see Perform Vector Laboratory Analyses).

The period of public health vigilance for mosquitoes (the duration of vector surveillance and control activities) should typically be at least 6 weeks after diagnosis or treatment of the person with the most recent malaria case (Table). This period accounts for the extrinsic incubation period and the lifespan of the mosquito but can vary depending on temperature and humidity conditions. If a new case is identified in a human, the mosquito vigilance period would start over.

#### Initiate Vector Control Activities

Vector control* should typically be implemented over a radius of at least 1 mile (1.6 km) around the site of suspected transmission as soon as possible to prevent ongoing malaria transmission by *Anopheles* mosquitoes. Strategies include actions that target adult mosquitoes and larvae (*33*). During outbreaks of mosquitoborne diseases such as malaria, the primary target of vector control should typically be adult female *Anopheles* mosquitoes that are potentially infective to humans, with the aim of reducing mosquito populations as quickly as possible (*34*). Adulticiding (i.e., vector control efforts aimed at killing adult mosquitoes) includes aerosol sprays (fogging) and residual sprays using chemical insecticides tailored to local mosquito populations. Aerosol spraying techniques can treat large areas with small amounts of pesticide and have been used safely for approximately 50 years. These aerosol sprays have been fully evaluated by the Environmental Protection Agency and pose minimal risks for humans or the environment when used according to the directions on the label (*35*). Aerosols are suspended droplets of insecticide that are effective only when the droplets come in direct contact with a mosquito during the short period (minutes) before the aerosol reaches the ground. Aerosols can be dispersed across wide areas after delivery from aircraft, trucks, or backpack sprayers. Aerosols are generally short-lived with droplets remaining in the air for ≤30 minutes depending on droplet size and wind conditions. Therefore, spraying should be timed to target adult female *Anopheles* mosquitoes when they are most active (generally at night), because the droplets have limited ability to penetrate vegetation and ensure insecticide exposure to resting mosquitoes. Aerosol sprays have no residual activity and can require several applications to achieve reductions in mosquito populations (*36*).

Residual sprays target potential resting sites of adult mosquitoes. This approach requires knowledge of the types of locations where mosquitoes are most likely to rest. Outside of the United States, the use of indoor residual spraying is commonly used to target malaria vectors which frequently enter houses and rest indoors. However, malaria vectors in the United States are generally less likely to enter and rest inside houses (*37*), particularly those of modern construction with screened windows and air conditioning which limit the entry of mosquitoes, making indoor residual spraying less effective and not routinely recommended in most U.S. settings. Outdoor residual spraying has been proposed to address malaria transmission in settings where malaria vectors feed and rest outdoors. Although intended to provide longer durations of efficacy compared with aerosol sprays, outdoor residual spraying can be logistically challenging to implement because outdoor resting sites of mosquitoes might not be known and insecticides sprayed outdoors can be degraded by sunlight or washed away by rain.

Adult control measures can be supplemented by larval source management (LSM), which targets the immature aquatic stages (eggs and larvae) of the mosquito life cycle. LSM includes habitat reduction through removal, treatment, or reduction of water bodies where adult mosquitoes lay eggs that develop into larvae that mature to a new brood of adult mosquitoes. However, although LSM eventually reduces the overall abundance of adult mosquitoes, it does not have an immediate effect on adult mosquito populations or disease transmission and should typically be considered as a supplementary intervention in response to outbreaks of vectorborne diseases. A wide range of larvicides and application methods are available for deployment. Similar to vector surveillance, the public health vigilance period for vector control efforts should typically continue for at least 6 weeks after the diagnosis or treatment of the patient with the most recent malaria case (Table).

#### Perform Vector Laboratory Analyses

The malaria outbreak investigation can be facilitated by knowing whether captured *Anopheles* demonstrate evidence of a recent blood meal from a malaria patient or if infective mosquitoes are present in the community. The former can be determined through detection of *Plasmodium* DNA in the abdomen of a captured mosquito, and the latter through detection of sporozoites in a mosquito’s salivary gland. *Anopheles* mosquitoes collected as part of the surveillance efforts described in this report could thus be tested by CDC to 1) determine whether mosquitoes have recently fed on an infected human host and 2) assess for infective stages (sporozoites) of the *Plasmodium* parasite (*38*). Assessing for mosquito infection might help characterize transmission potential in areas under surveillance. Microscopic examination of mosquito salivary glands is considered the gold standard for identifying sporozoites (i.e., the infective stage of the parasite), but this method is labor-intensive and time-consuming (*39*). Sporozoite stage–specific proteins can be identified using enzyme-linked immunosorbent assay (ELISA), but sensitivity might be limited, and this method is typically better suited for estimating sporozoite rates in malaria-endemic settings (*40*). Molecular methods such as PCR can also be used but are limited in capacity for differentiating infective (i.e., capable of transmitting the parasite) from infected (i.e., any stage of parasite present) mosquitoes (*41*). In a nonendemic outbreak context, this might not represent a limitation, because understanding whether a mosquito has fed on an infected human can aid in directing further vector and human case surveillance and control activities. Mosquito testing cannot exclude transmission because every mosquito in a given area cannot be captured. Because it often is not clear during the outbreak whether ongoing transmission is occurring, the number of mosquitoes needed to adequately detect transmission cannot be determined. CDC can assist with detection of *Plasmodium* DNA using molecular testing and sporozoite protein identification using a novel bead-based immunoassay demonstrated to have increased specificity over the traditional ELISA approach (Box 2) (*38*). Additional molecular analyses that might provide useful information, particularly if *Plasmodium* is detected in a mosquito, are blood meal source identification and *Anopheles* species identification (*42*–*44*).

### Enhanced Investigation Task C: Perform Molecular Surveillance of *Plasmodium* Parasites

After initial case confirmation, *Plasmodium* obtained from all locally acquired malaria cases should undergo molecular genotyping analysis to characterize strain relatedness and to interrogate sequence databases of well-characterized isolates from different global regions to infer probable geographic origin of the parasite. In addition, the genotypic data should be shared in public sequencing databases, allowing broader capability to identify other potentially related cases or clusters. Geographic-origin inference depends on the completeness and representativeness of the reference sequence database. Adequate pre-treatment whole blood might not always be available, some specimens might fail or yield partial data, and public database sharing should occur only after de-identification and in accordance with jurisdictional policy and data-sharing approvals. A shared genetic relationship between *Plasmodium* strains from malaria cases strengthens evidence regarding whether epidemiologic clusters (i.e., incident cases in time and space) are true outbreaks. Alternatively, the lack of a genetic relationship might refute an epidemiologic linkage; a temporospatial relationship might exist, although genotyping might implicate multiple genetically distinct strains. These data might indicate the presence of multiple unrelated clusters occurring at the same time and place, supporting that multiple separate malaria introduction events have occurred. Genotyping data should be interpreted alongside epidemiologic data and the laboratory context.

Although molecular genotyping for surveillance is indicated for all cases of autochthonous malaria, the best genotyping method employed for an investigation will vary depending on context. Factors such as the result of the initial PCR test (*45*), the type of specimen being tested (e.g., whole blood versus dried blood spot), the parasite density of the specimen, and the need for drug resistance testing can guide which assay should be selected. To address these factors, CDC employs various next generation sequencing (NGS) methodologies, including whole genome sequencing (*24*) and highly multiplexed NGS-based amplicon sequencing (*46*). Previously, health departments have engaged academic collaborators to assist with performing similar assays for locally acquired malaria. Fostering partnerships with state and Federal public health agencies in investigations is important because rapidly sharing sequencing findings might help link cases across jurisdictions. Health departments seeking molecular surveillance services from CDC can reach out directly (Box 2) for instructions on how to submit specimens and for other related inquiries. In contrast to CDC’s malaria diagnostics laboratory, molecular surveillance services offered by CDC can only be used for public health planning and decision-making because these tools have not been validated for use in direct patient care.

### Enhanced Investigation Task D: Conduct Community and Partner Outreach and Engagement

In the United States, mitigating the impact and potential poor outcomes from local malaria transmission requires outreach to 1) inform persons in the focal area of potential exposures and risks for illness; 2) provide awareness to the community on malaria symptoms, transmission, and when to seek care; and 3) educate and support local health care providers so that their patients can be promptly evaluated and treated for malaria. Health alerts (*47*,*48*) distributed to health care organizations in the days immediately after detection of an autochthonous case serve to educate health care providers on the diagnosis, treatment, and prevention of malaria. These alerts could also provide clinical resources for clinicians to obtain more information and training on malaria, such as CDC’s malaria clinical diagnosis and treatment guidance, CDC’s Malaria Hotline, and webinars (*17*). Clinicians could also be encouraged to report suspected and confirmed cases to health departments.

The public should also be engaged to help ensure awareness of the outbreak, the range of personal actions available to prevent mosquito bites (use EPA-registered insect repellent, wear loose-fitting long-sleeved shirts and pants, wear clothing and gear treated with permethrin, and control mosquitoes indoors and outdoors), and what to do if malaria symptoms develop. Public engagement can occur via spaces where persons congregate in large groups (e.g., community centers, schools, or places of worship), door-to-door canvassing, partnerships with community-based organizations, social media, or by reaching persons directly through reverse 911 calling (*49*). Persons could be encouraged to seek care if symptomatic and could be provided with resources on how to avoid malaria infection. Outreach materials should use plain language, be translated into languages appropriate to the local community, and be provided in accessible formats. Health departments should consider distributing topical repellent and other prevention materials (e.g., mosquito nets) to high-risk populations, such as persons experiencing housing instability or homelessness.

Public health officials could similarly engage local media outlets (television, radio, and newspapers) and social media to help broadcast public health messaging and education regarding malaria transmission, strategies for personal protection, and when and where to seek medical attention for malaria symptoms. In addition to providing public health guidance, public health officials could prepare responses for possible media inquiries during an outbreak. A list of possible media-related questions, examples of public health messaging, and resources are available (Appendix).

### Enhanced Investigation Task E: Determine if the Outbreak Has Ended

Health departments should consider when to officially declare the end of a malaria outbreak. For other infectious diseases, an outbreak is commonly declared to be over after two incubation periods without any cases; however, for malaria, the parasite life cycle needs to be considered (*8*,*50*). If no new locally acquired cases are identified during the 8 weeks after the diagnosis or treatment of the most recent local case, the likelihood of ongoing transmission is low. Malaria is not transmitted from female mosquitoes to their offspring (*51*). However, additional factors can dictate the end of the outbreak. These factors can include concern for delayed care-seeking or delayed diagnosis (which would extend the investigation period) and cold weather patterns (which might facilitate an earlier end point).

## Discussion

Preventing ongoing local malaria transmission depends on diligent human case–finding and treatment activities; however, limited evidence exists for the impact or effectiveness of these strategies in postelimination settings. WHO has conditional recommendations for reactive case detection and treatment in elimination and postelimination settings (*52*,*53*). Reactive case detection typically involves finding parasitemic persons by testing everyone living with or near an index case, or those potentially exposed to infection at the same time and place as the index case and providing treatment to those who receive a positive test result (*52*,*54*). Testing all persons is likely less useful in most U.S. settings because asymptomatic infection among most residents is expected to be less common than in endemic populations. In addition, no Food and Drug Administration–approved malaria rapid diagnostic test is currently available for self-use or general field use outside a clinical laboratory setting. Because malaria is potentially fatal, symptomatic persons who are suspected to have malaria should be referred to a health care facility for prompt clinical evaluation, laboratory testing, and treatment. Finally, CDC has not implemented other strategies such as mass drug administration, targeted or reactive drug administration, or chemoprophylaxis use for persons traveling to or living in an area in the United States where local cases have been identified since at least 1990 (*8*,*52*,*55*–*57*).

Preventing autochthonous malaria transmission also requires effective vector surveillance and vector control strategies (*53*). Vector control and surveillance have been limited by a lack of recent data to describe the distribution of *Anopheles* species in the United States (*4*,*7*). Previous maps illustrating the distribution of *Anopheles* species from a century ago require updating because of changes in mosquito habitat ecology over time. Routine mosquito surveillance and control at the jurisdictional level currently focuses on nuisance mosquitoes and arbovirus vectors in the *Aedes* and *Culex* genera. Thus, surveillance and control of most *Anopheles* mosquitoes in the United States are neither targeted nor optimized (*5*). Although efforts are underway, an ongoing need exists to better characterize the distribution of *Anopheles* species in the United States and validate control strategies for local *Anopheles* species. These control activities are resource-intensive, and coverage for the 6 weeks’ duration intends to target any infectious mosquitoes from the identified case, which is shorter than the time frame for the human case vigilance.

The United States has limited laboratory capacity to perform timely blood smear analysis for malaria diagnosis (*58*,*59*). Therefore, laboratory capacity should be rapidly assessed upon detection of a locally acquired malaria case and diagnostic reinforcement plans developed with health departments and CDC. Despite limitations with routine malaria diagnostic capacity in the United States, substantial advances have occurred in parasite molecular surveillance methods. NGS technologies have vastly improved upon previous *Plasmodium* genotyping methods (*24*,*46*). Genotyping methods based on NGS technologies can differentiate distinct malaria strains, provide information on their probable geographic origin, and characterize drug resistance profiles. However, the usefulness of molecular surveillance is contingent on remnant whole blood specimens from patients (with both imported and locally acquired malaria) being retained for reference laboratory confirmatory testing, as advised in CDC’s *Malaria Surveillance and Case Investigation Best Practices* (*20*). The analysis of genomic *Plasmodium* populations requires a molecular library of sequenced specimens from a temporally defined and known geographic origin, underscoring the need to conduct sequencing of domestic malaria specimens. Accurate exposure and travel history associated with these specimens is critical because the ability to infer the origin of a specimen requires reference sequences from strains of a similar geographic origin for comparison. Molecular surveillance data should be interpreted along with findings from the outbreak’s epidemiologic investigation, because ignoring this can result in misinterpretation of genotyping findings.

In contrast to vectorborne diseases that have other animal reservoirs, prompt detection and response efforts for malaria can successfully ensure that control measures directed at humans and mosquitoes effectively limit the spread of focal outbreaks. Reestablishment of malaria transmission (i.e., 3 consecutive years of at least three indigenous cases of the same *Plasmodium* species in the same geographic area) in the United States appears unlikely under current surveillance and response conditions, although focal outbreaks remain possible (*53*,*60*). The local malaria outbreaks that occurred in 2023 highlight the likelihood that limited focal transmission of this potentially life-threatening disease can still affect multiple communities and susceptible U.S. residents. Rapid identification of autochthonous cases by surveillance and implementation of interventions are important.

## Limitations

The public health activities listed in this report are subject to at least four limitations. First, limited data are available. Although a formal systematic review was not done, a thorough informal review of published and grey literature was conducted. The evidence base for preventing and responding to local malaria transmission in postelimination settings is limited (*53*). Published data on the comparative effectiveness of these recommended interventions are also limited, especially in a U.S. setting. Locally acquired mosquito-transmitted cases are rare, resulting in limited experiences of recent outbreak responses. Moreover, recent data are lacking describing the distribution of *Anopheles* vectors in the United States to guide surveillance and control activities. However, the recommendations here are consistent with what has been done historically for U.S. cases and with guidance from other non-endemic areas. Second, infrastructure, human resources, laboratory, and vector surveillance and response capacity vary across jurisdictions, which can affect the timeliness and comprehensiveness of investigations. Although asymptotic infection is unlikely among U.S. residents, there might be diagnostic delays and possible underascertainment of locally acquired malaria when it does occur. In addition, pre-treatment whole blood specimens for molecular linkage might not always be available. Third, operational thresholds in this guidance are based on a range of vector and parasite species biology and ecology to inform pragmatic programmatic planning within the United States. This guidance does not attempt to reflect the many subtleties known to influence the thresholds that depend upon specific malaria parasites and their vectors. Therefore, time and distance windows should be further tailored to specific conditions as needed. Finally, vector species composition, distribution, resting and feeding behavior, and movement patterns might change in the future.

## Conclusion

The United States remains susceptible to malaria reintroduction because of the continued importation of malaria cases and the continuous presence of competent mosquito vectors. In addition to a robust surveillance system, preventing reintroduction requires a multidisciplinary approach that encompasses epidemiologic, entomologic, and laboratory components. This updated guide provides a general framework for health departments to respond to autochthonous malaria outbreaks, and procedures might need to be adjusted depending on local context (e.g., local population demographics, ecologies, and vector biology). CDC continues to be available to assist local health departments prevent, detect, and respond to outbreaks of locally acquired mosquito-transmitted malaria.

## Footnotes

* All vector control measures must comply with Environmental Protection Agency (EPA) pesticide regulations and relevant state and local requirements, including permits, application methods, and community notifications. Local mosquito control programs, in coordination with state agencies, CDC, EPA, and community stakeholders are responsible for legal compliance, operational planning, and environmental safety.

## APPENDIX Malaria Messaging and Resources for Community Partners

### Examples of General Malaria Messaging

- Malaria is a mosquitoborne disease caused by a parasite. Persons with malaria often experience fever, chills, and flu-like illness. Severe complications and death can occur if left untreated.
- The United States reports approximately 2,000 cases of malaria each year. Most of these cases are travel-associated malaria (“imported” malaria) and occur in travelers returning from countries where malaria is still widespread (endemic).
- The *Anopheles* mosquitoes that transmit malaria are present across the United States. Areas with *Anopheles* mosquitoes and imported malaria cases are at higher risk for local malaria transmission.
- If a person in the United States already has malaria and is then bitten by an *Anopheles* mosquito, the mosquito can become infectious and could transmit the parasite to other persons in the area. The source of these locally acquired mosquito-transmitted malaria cases is likely related to an imported or travel-associated case of malaria.
- The most effective way to prevent malaria in the United States is to prevent mosquito bites and ensure early diagnosis and treatment of imported cases. Travelers to malaria-endemic areas should take recommended steps to prevent acquiring malaria while traveling, including taking medications to prevent malaria.

### Potential Media Inquiry Questions

- When was the last time locally acquired (mosquito-transmitted) malaria was detected in my area or the United States?
- Should we (the community) be concerned?
- Where was the recent case?
- What can I do to prevent getting locally acquired (mosquito-transmitted) malaria? (How does it spread?)
- What are you doing to prevent the spread of locally acquired (mosquito-transmitted) malaria?
- What should someone do if they think they have malaria?
- How can we ensure the blood supply is safe?

### Examples of Media Press Releases

- Florida Department of Health Issues Mosquito-Borne Illnesses Advisory | Florida Department of Health
- Maryland Department of Health Announces Positive Case of Locally Acquired Malaria | Maryland Department of Health
- Locally Acquired Malaria Identified in Arkansas | Arkansas Department of Health

### Clinician Education and Outreach Resources

- Locally Acquired Malaria Cases Identified in the United States | CDC Health Alert Network
- Important Updates on Locally Acquired Malaria Cases Identified in Florida, Texas, and Maryland | CDC Health Alert Network
- Malaria 101 for the Healthcare Provider | CDC (Continuing Medical Education, Continuing Nursing Education, and Continuing Education Units are available)
- Review of Malaria Diagnosis and Treatment in the United States | CDC
- Clinical Guidance: Malaria Diagnosis & Treatment in the U.S. | CDC

### Community Outreach Resources

- Malaria is a Serious Disease | CDC
- Malaria is a Serious Disease (in Spanish) | CDC
- Preventing Mosquito Bites | CDC

### Locally Transmitted Malaria Outbreak Reports

- Outbreak of locally acquired mosquito-transmitted (autochthonous) malaria — Florida and Texas, May–July 2023 | CDC
- Notes from the field: locally acquired mosquito-transmitted (autochthonous) *Plasmodium* *falciparum* malaria — National Capital Region, Maryland, August 2023 | CDC
- Locally acquired (autochthonous) mosquito-transmitted *Plasmodium vivax* malaria — Saline County, Arkansas, September 2023 | CDC
- Locally acquired mosquito-transmitted malaria: a guide for investigations in the United States | CDC
- Public health response to the first locally acquired malaria outbreaks in the US in 20 years | JAMA Network
- Local transmission of *Plasmodium vivax* malaria — Palm Beach County, Florida, 2003 | CDC
- Local transmission of *Plasmodium vivax* malaria — Virginia, 2002 | CDC
- Probable locally acquired mosquito-transmitted *Plasmodium vivax* infection — Suffolk County, New York, 1999 | CDC
- Changing patterns of autochthonous malaria transmission in the United States: a review of recent outbreaks | CDC
